# Non-specific Effects of Live Attenuated Pertussis Vaccine Against Heterologous Infectious and Inflammatory Diseases

**DOI:** 10.3389/fimmu.2018.02872

**Published:** 2018-12-07

**Authors:** Stéphane Cauchi, Camille Locht

**Affiliations:** ^1^Univ. Lille, U1019, UMR 8204, CIIL–Centre for Infection and Immunity of Lille, Lille, France; ^2^CNRS UMR8204, Lille, France; ^3^Inserm U1019, Lille, France; ^4^CHU Lille, Lille, France; ^5^Institut Pasteur de Lille, Lille, France

**Keywords:** *Bordetella*, influenza, RSV, asthma, contact dermatitis, inflammation

## Abstract

*Bordetella pertussis* is the agent of pertussis, also referred to as whooping cough, a disease that remains an important public health issue. Vaccine-induced immunity to pertussis wanes over time. In industrialized countries, high vaccine coverage has not prevented infection and transmission of *B. pertussis*, leading to periodic outbreaks in people of all ages. The consequence is the formation of a large source for transmission to children, who show the highest susceptibility of developing severe whooping cough and mortality. With the aim of providing protection against both disease and infection, a live attenuated pertussis vaccine, in which three toxins have been genetically inactivated or removed, is now in clinical development. This vaccine, named BPZE1, offers strong protection in mice and non-human primates. It has completed a phase I clinical trial in which safety, transient colonization of the human airway and immunogenicity could be demonstrated. In mice, BPZE1 was also found to protect against inflammation resulting from heterologous airway infections, including those caused by other *Bordetella* species, influenza virus and respiratory syncytial virus. Furthermore, the heterologous protection conferred by BPZE1 was also observed for non-infectious inflammatory diseases, such as allergic asthma, as well as for inflammatory disorders outside of the respiratory tract, such as contact dermatitis. Current studies focus on the mechanisms underlying the anti-inflammatory effects associated with nasal BPZE1 administration. Given the increasing importance of inflammatory disorders, novel preventive and therapeutic approaches are urgently needed. Therefore, live vaccines, such as BPZE1, may offer attractive solutions. It is now essential to understand the cellular and molecular mechanisms of action before translating these biological findings into new healthcare solutions.

## Introduction

Despite of the use of efficacious vaccines, *Bordetella pertussis* is still one of the leading causes of neonatal morbidity and mortality worldwide (1). In the 90s, acellular pertussis vaccines (aPV) have been increasingly replacing the first-generation, whole-cell vaccines (for the description of pertussis vaccines, see Table 1) (2, 3). In countries with high aPV vaccination coverage, the resurgence of pertussis has revealed that aPV-induced immunity decreases faster than that induced by whole-cell vaccines or by natural infection (1). Furthermore, aPV vaccination skews the immune response to a Th2 type, both in mice and in human infants (4–6). Given that immunity induced by *B. pertussis* infection decreases later than vaccine-induced immunity (7), and that newborns are capable of inducing a strong Th1 response upon infection (8), a live attenuated pertussis vaccine candidate has been developed to be administered by nasal inoculation (9). Named BPZE1, this vaccine candidate has successfully completed a phase I clinical trial in humans (10). Genetic modifications were made to remove or inactivate three major toxins, pertussis toxin (PTX), tracheal cytotoxin (TCT), and dermonecrotic toxin (DNT). A single intranasal administration of BPZE1 led to strong and prolonged B cell and Th1 T cell responses, inducing protection against challenge infection (9, 11–14). A single nasal administration of BPZE1 in infant mice was associated with stronger protection than that induced by two inoculations of aPV (9) and was lasting substantially longer (13, 14). In addition to mice, juvenile baboons were protected by a single nasal administration of BPZE1 against infection and whooping cough disease upon challenge with a highly virulent *B. pertussis* clinical isolate (15). Therefore, BPZE1 is now in further clinical development as a vaccine candidate against pertussis in adults and in neonates.

**Table 1:** Vaccine formulations against pertussis.

**Vaccine**	**Producer**	**PTX (μg)**	**FHA (μg)**	**PRN (μg)**	**FIM (μg)**
**DTaP**					
Infanrix	GlaxoSmithKline	25	25	8	–
Boostrix	GlaxoSmithKline	8	8	2.5	–
Daptacel	Sanofi Pasteur	10	5	3	5
Adacel	Sanofi Pasteur	2.5	5	3	5
**DTwP**					
D.T.COQ/D.T.P.	Sanofi Pasteur	NA	NA	NA	NA
Triple Antigen	Serum Institute of India Ltd.	NA	NA	NA	NA
Quinvaxem	Crucell-Janssen	NA	NA	NA	NA
**LIVE ATTENUATED VACCINE**					
BPZE1 (Phase II)	ILiAD Biotechnologies	–	NA	NA	NA

## Heterologous protection by BPZE1

Clinical, immunological, and epidemiological studies have shown that live vaccines can induce immunity to organisms other than those against which they were initially intended. The Bacillus Calmette-Guerin (BCG), smallpox, measles, oral polio, and yellow fever vaccines have been extensively documented to decrease disease and/or mortality from infections that are different from tuberculosis, smallpox, measles, polio, yellow fever, respectively (16). When repurposed against cancer, inflammatory and/or auto-immune disorders, promising effects have been observed for some of these vaccines (17). These heterologous non-specific effects, also termed “off-target effects,” seem to be limited to live vaccines (18). Therefore, the heterologous protection by BPZE1 was also extensively investigated.

In addition to *B. pertussis*, BPZE1 protects mice also against lung infection by other *Bordetella* species, although this was not observed after vaccination with aPV (9, 12, 19). *Bordetella parapertussis* is a respiratory pathogen that causes chronic pneumonia in sheep or pertussis-like disease in humans, albeit usually less serious than pertussis caused by *B. pertussis*. A single nasal BPZE1 vaccination led to strong protection against colonization by *B. parapertussis* (9) and protection could be transferred by splenocytes but not by serum of BPZE1-vaccinated mice, whereas serum from convalescent mice was able to protect against re-challenge with *B. parapertussis*. These observations indicate that BPZE1-mediated cross-protection was cell-mediated (12). *Bordetella bronchiseptica* can infect a large variety of mammalian species, including humans, and can cause mild to severe cough. In a mouse lethal challenge model, BPZE1 reduced both death and lung colonization induced by *B. bronchiseptica* (19). Interestingly, these protective effects depend on two distinctive mechanisms. The decrease in colony-forming units (CFUs) in the lungs relied on adaptive T-cell-mediated immunity. However, protection against mortality was primarily due to BPZE1 potent anti-inflammatory properties. Compared to non-vaccinated mice, BPZE1-vaccinated animals had reduced inflammation, neutrophil infiltration and tissue damage in the lungs upon *B. bronchiseptica* infection. Nasal vaccination with BPZE1 also primed mice for the induction or recruitment of CD4^+^CD25^+^FoxP3^+^ regulatory T cells in the lungs the amounts of which strongly increased upon *B. bronchiseptica* challenge only in the BPZE1 vaccinated mice. The role of these cells in the anti-inflammatory activities of BPZE1 was evidenced by the significantly decreased protection against *B. bronchiseptica*-induced mortality when they were depleted using anti-CD25 antibodies 24 h before challenge.

The above observations suggest that the heterologous protection induced by BPZE1 against the closely related pathogens *B. parapertussis* and *B. bronchiseptica* is likely due to cross-reactive B- or T-cells. Even the anti-inflammatory protective activity against *B. bronchiseptica*-induced mortality may be mediated by cross-reactive CD4^+^CD25^+^FoxP3^+^ regulatory T cells. However, heterologous protection elicited by BPZE1 has also been assessed against very distant pathogens, totally lacking any cross-reactive antigens at the B- or T-cell levels, such as highly pathogenic influenza A virus (20). A single nasal inoculation of BPZE1 decreased mortality caused by a virulent mouse-adapted Influenza A strain (20). When mice were infected with 2 LD_50_ of a mouse-adapted H3N2 virus 6 or 12 weeks after a single BPZE1 vaccination, 60% of them survived the viral challenge. No protection against H3N2-induced death was observed when the vaccine was heat-inactivated, or when it was given 3 weeks prior to challenge. These data suggest that only live BPZE1 provides protection and that BPZE1-mediated protection takes several weeks to be established for a long period of time. Protection was dose-dependent and required at least 5 × 10^6^ CFU of BPZE1 in this model. Although live BPZE1 protected against H3N2-induced death, it did not significantly reduce the viral load, demonstrating that the virus particles were not directly targeted by the protective mechanism. This observation is consistent with the lack of B- or T-cell cross-reactivity between BPZE1 and the virus. However, BPZE1 vaccination decreased lung immunopathology, decreased neutrophil and increased macrophage numbers in the bronchoalveolar lavage fluids. Furthermore, BPZE1 protected against lymphocyte depletion and reduced the inflammatory cytokine storm resulting from the viral infection, as evidenced by a decrease in IL-1β, IL-6, and GM-CSF as compared to the non-vaccinated mice upon viral challenge. Strikingly, an additional administration of BPZE1 improved survival of the influenza-infected mice and further decreased the inflammatory cytokine levels. Since this protective mechanism did not rely on adaptive immunity, these observations suggest that BPZE1 could induce trained innate immunity, which has been shown to be based on epigenetic reprogramming of monocytes (21). This may lead to transcriptional programs that rewire the intracellular immune signaling of these innate immune cells but also induce a shift of cellular metabolism, thus increasing the innate immune cells' capacity to respond to stimulation. Although not yet studied, administration of BPZE1 may potentially be associated with specific epigenetic events that are known to control myeloid cell differentiation, the acquisition of myeloid identity and innate immune memory (22).

However, the mechanism of the protective anti-inflammatory effect of BPZE1 is not yet known. Although BPZE1 administration resulted in a transient increase of IL-10-producing CD4^+^ T cells in the bronchoalveolar lavages (23), the role of IL-10 in BPZE1-mediated protection against influenza remains uncertain. TGF-ß levels did not differ between vaccinated and non-vaccinated mice. Whether CD4^+^CD25^+^FoxP3^+^ regulatory T cells play a role in this model was not investigated.

Protection against viral diseases has also been demonstrated in a murine model of respiratory syncytial virus (RSV) infection (23). RSV usually does not cause death in mice but induces dose-dependent weight loss. When mice were vaccinated with BPZE1 14 days before RSV infection, the weight loss was completely abolished. Compared to the non-vaccinated mice, the viral load was reduced 2- to 3-fold in the vaccinated animals, despite the lack of cross reactivity between BPZE1 and RSV. However, this does not fully account for the protective effect against weight loss. Lymphocyte recruitment to the lungs after RSV infection was also significantly reduced in the vaccinated mice, whereas the amounts of macrophages and polymorphonuclear cells in the bronchoalveolar lavages were increased in the BPZE1-treated animals. Interestingly, neonatal vaccination with BPZE1 induced protection for a long period of time against RSV disease as a single nasal BPZE1 dose administered to 2–5-day old mice significantly decreased RSV-induced weight loss when they reached adulthood.

Interestingly, prior BPZE1 vaccination led to an increase in IL-10-producing CD4^+^ T cells after RSV infection, whereas the numbers of IFN-γ-producing cells were reduced. This is in apparent contrast to what was reported in the influenza model, where BPZE1 treatment resulted in reduced levels of both IL-10 and IFN-γ in the bronchoalveolar lavages after viral challenge (20). Whether this merely reflects different read-outs or timings between the two studies or whether it suggests different mechanisms of protection against the two diseases remains to be investigated. In the RSV model, the numbers of virus-induced IL-17-producing CD4^+^ T cells were also increased by prior BPZE1 vaccination (23), while TNF-α and RANTES levels in the bronchoalveolar lavages were decreased. The role of IL-17 in BPZE1-mediated protection against RSV disease could be demonstrated by the use of blocking anti-IL-17 antibodies administered before and during challenge to the BPZE1-vaccinated mice. Administration of these antibodies reestablished the weight loss prevented by BPZE1 vaccination and prevented the recruitment of IL-10/IFN-γ double positive T cells, whereas it did not increase viral load. These data indicate that BPZE1-mediated protection against RSV disease does not merely rely on its ability to slightly decrease the viral load. IL-17 may be important for the recruitment or expansion of IL-10 producing T cells that in turn may decrease lung inflammation. The role of IL-10 in limiting RSV disease has been well documented (24, 25). However, in addition to the induction of IL-17- and of IL-10-producing T cells, BPZE1 vaccination prior to RSV challenge also induced elevated levels of CD4^+^FoxP3^+^ regulatory T cells, which have also been documented to modulate RSV disease (26). The role of these cells in BPZE1-mediated protection against RSV disease has not been investigated yet.

## Protection Against Non-Infectious Diseases by BPZE1

As BPZE1 provided protection against inflammation induced by viral infections, it was also investigated whether BPZE1 can prevent inflammation caused by non-infectious etiologies, such as allergen-induced asthma. In a murine asthma model, nasal inoculation of BPZE1 10 days before ovalbumin sensitization reduced peribronchial inflammation upon ovalbumin challenge, as compared with the non-vaccinated control group of sensitized mice (27). In contrast, nasal infection with virulent *B. pertussis* prior to ovalbumin sensitization exacerbated the pathology. This observation is in line with *B. pertussis*-caused exacerbation of asthma in humans (28) and with the recently developed notion that *B. pertussis* infection may also be an important cause of asthma in humans (29). In contrast to virulent *B. pertussis*, which exacerbated goblet cell hyperplasia and mucus secretion, nasal administration of BPZE1 reduced mucus secretion in the ovalbumin-sensitized mice and reduced bronchial hyperreactivity (27). This was paralleled by a reduced inflammatory infiltration of the airways, as evidenced by a reduced total cell number, neutrophils and especially eosinophil influx, upon aerosol ovalbumin challenge in the BPZE1-treated mice compared to the non-vaccinated mice. However, BPZE1 treatment did not decrease the ovalbumin-specific serum IgE responses, whereas infection with virulent *B. pertussis* increased the ovalbumin-specific serum IgE levels. BPZE1 administration before sensitization by ovalbumin also decreased the levels of the Th2 cytokines IL-4, IL-5, and IL-15 in the bronchoalveolar lavages, whereas it significantly increased the IFN-γ levels. Thus, since BPZE1 vaccination had no effect on IgE production, it is tempting to hypothesize that the increased IFN-γ production in BPZE1-vaccinated mice has antagonized Th2-driven fibrosis and remodeling of the airways through eosinophil recruitment (30).

When the mice were vaccinated with BPZE1 6 weeks before sensitization, comparable findings were reported (31), indicating that the protective effect of BPZE1 is long-lasting. Again, pre-treatment with BPZE1 protected against airway pathology, whereas pretreatment with virulent *B. pertussis* exacerbated it, even after total clearance of the *Bordetella* infection. Similarly, mucus hypersecretion induced by ovalbumin was markedly decreased in BPZE1-treated mice, as was the inflammatory cell recruitment in the lungs, especially the recruitment of eosinophils. Interestingly, in contrast to the study by Kavanagh et al. (27), BPZE1 treatment also significantly reduced total and ovalbumin-specific serum IgE responses in ovalbumin sensitized and challenged mice (31), although the reduction was < 2-fold. Finally, the levels of the Th2 cytokines IL-4, IL-5, and IL-13 in the bronchoalveolar lavages were also decreased in the BPZE1-vaccinated mice, as were the levels of IL-1ß and IL-2, whereas the IFN-γ levels did not change. Interestingly, in this model, bronchoalveolar IL-10 levels were increased in the ovalbumin-treated mice as compared to the controls, and this increase was abolished by prior BPZE1 administration. Thus, it is possible that the protective effect of BPZE1 in this model does not depend on IL-10, although this remains to be investigated. Overall, these studies show that BPZE1 is a potent immunomodulatory agent able to suppress allergic asthma in mice even several weeks after a single administration, which is different from most other anti-inflammatory agents that only provide short-term effects.

As in the asthma models described above nasal BPZE1 administration also affected serum antibody responses (31), as well as T-cell responses in the spleen (31), it is possible that its off-target effects may not be restricted to the respiratory tract. In a murine model of allergic contact dermatitis, nasal immunization with BPZE1 was indeed found to reduce dinotrochlorobenzine-induced ear swelling and inflammation of the skin (31). When mice were intranasally vaccinated twice with BPZE1 at a 4-week interval and then treated with dinitrochlorobenzene, a significant prevention of ear swelling was observed, with decreased tissue edema, inflammatory cell infiltration and local production of pro-inflammatory cytokines. However, in contrast to the allergic asthma model, a single administration of BPZE1 did not significantly reduce ear swelling and inflammatory cell infiltration of inflammatory cells in the skin, and two doses were necessary. Two intranasal doses of BPZE1 also reduced the amounts of IL-1ß, IL-2, IL-17, IL-6, TNF-α, and IL-4 in ear homogenates of dinotrochlorobenzine-treated mice, without affecting the levels of IL-10. Thus, intranasal BPZE1 treatment is associated with a systemic protection against inflammatory disorders both at local and at distant sites.

## Possible Mechanisms Underlying the Anti-Inflammatory Effects of BPZE1?

Although BPZE1 is associated with potent anti-inflammatory properties, it is likely that its nasal administration initially induces mild inflammation, since it is able to induce T and B cell responses to *B. pertussis* antigens. However, this moderate inflammation appears to be well controlled and rapidly resolved. Little is known about the post-vaccination resolution of inflammation. It has been hypothesized that there is a spontaneous decay of proinflammatory signals, potentially helped by cells of the immune system. Specifically, the regulatory T cells, which are the gateway cells protecting hosts from autoimmunity (32) and which dampen the immune response back to homeostatic levels after an acute reaction (33, 34). Regulatory T cells and Th17 cells have a dynamic relationship between immunity and inflammation, as both are linked with tolerance and immunosuppression (35). Therefore, a modification of the delicate balance between subsets of regulatory T cells and effector T cells in BPZE1-treated mice may result in a short-term mild inflammatory response followed by an tolerogenic response in case of subsequent immune stimulation.

However, as of today the cellular and molecular mechanisms of the BPZE1-associated anti-inflammatory effects remain unclear. Both CD4^+^CD25^+^FoxP3^+^ Treg cells and IL-10-producing CD4^+^ T cells may depend on IL-17 and be involved in a synergistic manner (36). However, this may vary between disease models. *In vitro* studies on human dendritic cells suggest that BPZE1 can drive Th1/Th17 responses in humans (37). BPZE1-treated dendritic cells induced T lymphocytes expressing CD39/CD73 to generate adenosine using ATP as substrate, and CD38/CD203a/CD73, which can hydrolyze NAD+ to generate adenosine as well (38), and adenosine is known for its anti-inflammatory properties (39). Thus, the induction of these enzymes may result in a regulatory phenotype that may contribute to the mechanism underlying the anti-inflammatory properties of BPZE1. Interestingly, the anti-inflammatory activities of BPZE1 are neither associated with immunodeficiency, since antibody levels or antigen-specific T cell responses to viral or bacterial antigens are not changed by BPZE1 inoculation, nor with a rise in bacterial (9) or viral load (20, 23) upon heterologous infection.

Whereas, many pathogenic bacteria lead to inflammation in the host, some bacterial proteins have the ability to prevent inflammatory responses in order to increase their survival within the host. Pathogens have developed different strategies to counter inflammatory mechanisms, such as escape from the host defense, inhibition of leukocyte recruitment to an inflamed area, deactivation of anti-microbial peptides, increased stability of endogenous inflammatory inhibitors, increased expression of anti-inflammatory cytokines, and NF-κB pathway inhibition through the cleavage of p65/relA (40).

The initial encounter of pathogens with the immune system occurs in an environment often conditioned and regulated by its endogenous microbiota. Thus, it may also be possible that the anti-inflammatory effects observed after BPZE1 administration is partly driven by commensals. It is known that commensals are critical and active inducers of regulatory responses. For example, induction of regulatory T cells was proposed as one of the main mechanisms of action of probiotics—defined bacteria known to confer a health benefit to the host (41). Whether BPZE1 administration alters the endogenous microbiota has not yet been investigated.

Three virulence factors are lacking or are inactivated in BPZE1, compared to the virulent parental strain. The DNT gene is deleted in BPZE1, the PTX gene has been genetically modified, which results in an enzymatically inactive molecule, and the level of TCT has been reduced to background levels. When functionally active, these virulence factors exacerbate airway inflammatory responses during *B. pertussis* infection (42–44). Therefore, their loss in BPZE1 would be expected to abolish the inflammatory effects.

The lack of widespread occurrence of PTX-deficient strains in the acellular vaccine era suggests that this virulence factor is crucial for *B. pertussis* pathogenicity and/or transmission (45). A *B. pertussis* strain not producing PTX failed to induce lethality in 4-week-old young mice, and to effectively colonize the airways of infected mice (46). A U.S. *B. pertussis* isolate lacking both PTX and pertactin (PRN) (47) caused no disease in a non-human primate model of pertussis (45). Mice infection with high doses of PTX-deficient strains promptly resolved inflammatory airway pathology, whereas infection with isogenic PTX-producing strains significantly prolonged inflammatory events and airway pathology (42, 48). Similarly, mice infected with a PTX-producing *B. pertussis* strain have significantly exacerbated respiratory reflex responses after intratracheal inoculation of bradykinin, compared to mice infected with a PTX-deficient strain (49, 50). Paroxysmal coughing lasting several days in rats infected with virulent *B. pertussis* (51) was not observed in rats infected with a PTX-deficient strain (52). Expression of inflammatory cytokine and chemokine genes was increased in *B. pertussis*-infected mouse lungs, but not in mice infected with a PTX-deficient strain (42, 53). Two distinct signaling mechanisms are used by PTX to subvert cellular responses: ADP-ribosylation of the Gα_i/o_ proteins by the A-protomer of the toxin (G_i/o_ protein-dependent action) and the interaction of the B-oligomer with cell surface proteins (G_i/o_ protein-independent action) (54). As BPZE1 produces enzymatically inactive PTX, it is likely that the absence of inflammation upon BPZE1 administration is due to the inactivation of this enzymatic activity.

However, the absence of inflammatory properties does not explain the potent anti-inflammatory properties of BPZE1, which are likely due to *B. pertussis* factors that are yet to be discovered. The broad effects of PTX on cell signaling may interact with these unknown factors and thus mask specific immunomodulatory properties.

The adenylate cyclase toxin (ACT), still present in BPZE1, is one potential candidate. This toxin induces a fast upregulation of cellular cAMP levels, which inhibits certain antibacterial activities, such as reactive oxygen species production, phagocytosis, and oxidative burst induction in the neutrophils (55–59). During early infection, inhibition of these activities abolishes innate immune control of *B*. *pertussis* (60, 61). In epithelial cells and macrophages, ACT may promote infection by influencing the secretion of cytokines and chemokines (43). Purified ACT was shown to suppress the generation of IL-12 and TNF-α and to increase the production of IL-6 and IL-10 in human monocyte-derived dendritic cells (MDDC) and macrophages activated by lipopolysaccharide (LPS) (61–65), implying that ACT activity is associated with a down-regulation of inflammation. In human bronchial epithelial cells, it was reported that *in vitro* ACT activity was related to the inhibition of expression of genes coding for the proinflammatory cytokines IL-1β, TNF-α and IL-8 (66). Conversely, within 1 h of toxin addition, the activity of ACT resulted in increased expression of genes coding for IL-1α, IL-6, and IL-10. However, within 24 h after the addition of ACT, the expression levels of these genes were back to the basal state (66). Interestingly, co-incubation of ACT (10 ng/ml) and LPS led to survival signaling in MDDCs and bone-marrow-derived dendritic cells (BMDCs) (67). ACT committed TLR-stimulated dendritic cells to induce CD4^+^CD25^+^Foxp3^+^T regulatory cells *in vitro*. In mice, ACT-deficient mutants of *B. pertussis* are impaired in their ability to infect, but this impairment was detectable at later time points than that seen with a PTX-deficient strain (68). These data are consistent with another study reporting that the ACT gene promoter was upregulated later than the PTX gene promoter after infection (69). Therefore, in virulent *B. pertussis*, PTX may act early during infection to suppress neutrophil influx and ACT may act afterwards to affect neutrophils and other cells recruited at the site of infection (68). In the absence of active PTX early activation of ACT may thus strongly inhibit inflammatory responses in BPZE1-treated hosts.

Recently, ACT was found to interact with filamentous haemagglutinin (FHA) to suppress *in vitro* production of a biofilm (70). An immune-regulatory role of FHA has been proposed, as microbe-specific type 1 regulatory T cell (Tr1) clones specific for FHA could be generated from the lungs of mice during acute infection by *B. pertussis* (71). The Tr1 clones secreted high levels of IL-10 (but not IL-4, nor IFN-γ), expressed T1/ST2 and CC chemokine receptor 5 and inhibited Th1 responses. In addition, FHA suppressed IL-12 and stimulated IL-10 generation by dendritic cells, which directed naive T cells toward the regulatory subtype. However, another recent study did not confirm the production of IL-10 by DCs upon FHA treatment (72). Nevertheless, *in vivo* systemic administration of FHA was shown to suppress pro-inflammatory cytokine and to enhance anti-inflammatory cytokine generation by innate immune cells. They suppressed (directly or indirectly) intestinal inflammation in a T cell-mediated model of colitis through the generation of regulatory T cells (73). The inhibitory role played by FHA was also observed in a *B. bronchiseptica* animal model of infection (43). A first analysis demonstrated that FHA can down-regulate the innate immune response against *B. bronchiseptica* infection, leading to lessened inflammation and longer bacterial persistence (74). In the same model, additional studies reported that without FHA, *B. bronchiseptica* triggers a Th17 response leading to fast bacterial clearance, while the wild-type strain expressing FHA caused persistent infection (58).

PRN is yet another candidate potentially endowed with anti-inflammatory properties. PRN-deficient *B. pertussis* strains induced stronger TNF-α, IL-6, IL-8, and G-CSF production when incubated with human DCs than their PRN-producing isogenic counterparts (75). Furthermore, the expression of *IRAK1/2, JUN* and *MAP2K3*, as well as that of *TLR1, TLR5*, and *TLR6* was significantly more up-regulated in human DCs when incubated with PRN-deficient *B. pertussis* than with PRN-producing *B. pertussis*. *In vivo*, infection of mice with PRN-deficient *B. pertussis* also resulted in increased serum levels of some cytokines, such as TNF-α, IL-8 G-CSF, and IL-1ß, compared to infection with PRN-producing *B. pertussis*. In the lungs of mice the genes involved in lipid release, necrosis and cell death were significantly more expressed after infection with PRN-deficient *B. pertussis* than with PRN-producing *B. pertussis*.

Obviously, many questions remain to be answered before a vaccine such as BPZE1 can be considered as a tool to prevent or treat heterologous inflammatory diseases. Whether any of these factors alone or in combination may account for the anti-inflammatory properties of BPZE1 obviously requires further studies. Monitoring immune cell recruitment over time in bronchoalveolar lavage fluids and examining gene expression profiles of each cell type after BPZE1 treatment may improve our understanding. Inactivation or suppression of additional factors in BPZE1 may also be necessary to identify the causative factors of the anti-inflammatory effects. It is also unknown how important lung colonization with live BPZE1 is, whether BPZE1 induces dysbiosis in the respiratory tract or elsewhere, and whether this might play a role. The duration of the anti-inflammatory effect has not been explored either. It will also be important to examine whether a single mechanism is responsible for the anti-inflammatory properties in all models investigated so far, or whether different mechanisms might be at play according to the model. Finally, all evidence for heterologous protection mediated by BPZE1 has been obtained in murine models and it remains to be assessed whether BPZE1 expresses its anti-inflammatory properties also in other species, including humans. These are among the questions that should be addressed in future investigations.

## Conclusion

Vaccination is considered as one of the most successful and cost-effective medical interventions ever introduced (76, 77). The assumption that vaccines have non-specific effects was first reported in the 1990s at the Bandim Health Project in West Africa by Aaby et al. (78). Since then, our understanding of the immunological landscape is changing drastically. Beyond its capacity to protect against *B. pertussis* infection, the protection of BPZE1 against heterologous infections and inflammatory diseases make this live attenuated vaccine a good candidate to treat a variety of diseases associated with exacerbated inflammation. Given the generic mechanisms and robustness of innate immunity, the capacity to understand and take advantage of this very effective system supplies an attractive method to counteract infections (79). Analyzing how pathogens induce anti-inflammatory and anti-immune machineries in their hosts will lead to a better identification of the different defense weaknesses and thereby more accurately decipher the fundamental mechanisms of microbial pathogenesis (80). Given the rising rate of new infectious and inflammatory diseases and the persistence of classical infections, including pertussis, this research remains essential to consider new preventative and therapeutic strategies.

## Author Contributions

All authors listed have made a substantial, direct and intellectual contribution to the work, and approved it for publication.

### Conflict of Interest Statement

The institution employing CL (Inserm) holds several patents on the BPZE1 vaccine, and CL is a consultant of ILiAD Biotechnologies, the company that has licensed these patents. The remaining author declares that the research was conducted in the absence of any commercial or financial relationships that could be construed as a potential conflict of interest.
